# Utilization of Head Imaging in Children and Adolescents With First-Episode Psychosis: A Retrospective Analysis

**DOI:** 10.7759/cureus.65675

**Published:** 2024-07-29

**Authors:** Vandana Doda, Archana Kumar, Shaina Schwartz

**Affiliations:** 1 Psychiatry, Moses Cone Hospital, Greensboro, USA; 2 Clinical Sciences, Fred Wilson School of Pharmacy, High Point University, High Point, USA

**Keywords:** computed tomography, first-episode psychosis, child and adolescent psychiatry, magnetic resonance imaging, head imaging

## Abstract

Background

Psychotic disorders are commonly diagnosed in the mid-20s but symptoms often emerge earlier during late teenage years to mid-20s. Notably, studies have shown that psychotic symptoms can also affect younger individuals, with a higher prevalence among preteens than teens. Head imaging via computed tomography (CT) or magnetic resonance imaging (MRI) can be performed to rule out non-psychiatric causes of psychotic symptoms in this population but may pose additional risks and financial burdens. Practice patterns vary regarding when to utilize head imaging in pediatric patients with first-episode psychosis (FEP). The purpose of this study is to better understand the use of head imaging in pediatric FEP and associated patient characteristics.

Methods

A retrospective cohort study was performed. Eligible patients were <18 years of age with an encounter documented between 2013 and 2023 where a diagnosis code for psychosis was first applied. Medical records were manually reviewed if head imaging was performed during the index encounter or within one month. Descriptive statistics were used to report the study population demographics. Independent t-testing was used to compare characteristics between patients who did and did not receive head imaging.

Results

A total of 113 patients met the inclusion criteria for the study, of which 12 (10.6%) received head imaging within the specified timeframe. All received CT criteria head scans, and a significantly higher proportion were African American or Black when compared to those who did not receive head imaging (10/12 (83.3%) vs. 53/101 (52.5%) p=0.023). None of the imaging tests performed yielded significant neurological findings that suggested an underlying pathology for psychosis.

Conclusions

Head imaging was rarely utilized for the initial assessment of pediatric FEP in this study. When it was used, CT head scans were the modality of choice but did not yield any remarkable findings to suggest a non-psychiatric cause of psychotic symptoms. This adds to the body of evidence supporting a conservative approach when considering head imaging in pediatric FEP.

## Introduction

Psychosis manifests as disruptions in thought, perception, and behavior resulting in a loss of reality testing. Psychotic disorders are typically diagnosed in early adulthood (median age of 25 years at onset) but symptoms often emerge earlier during the late teenage years to mid-20s [1]. However, epidemiological studies have revealed that psychotic symptoms can also occur in younger populations, with notable median prevalence rates of 17% among nine- to 12-year-olds and 7.5% among 13- to 18-year-olds [2]. A comprehensive evaluation, including history, mental status exam, complete physical and neurological examination, and routine laboratory testing, is crucial to rule out any underlying metabolic (electrolyte abnormalities, thyroid disorder, etc.), neurological (tumors, seizures, migraine, etc.), or infectious etiologies (HSV, HIV, toxoplasmosis, syphilis, Lyme disease, etc.). Although head imaging in the form of a computed tomography (CT) scan or magnetic resonance imaging (MRI) is often included as a part of the initial workup, its routine use for the assessment of child and adolescent patients with first-episode psychosis (FEP) is controversial and not recommended by the American Academy of Pediatrics (AAP) due to limited evidence [3]. While missing an organic etiology can worsen prognosis, some clinicians advise against routine head imaging in pediatric FEP in the absence of neurological deficits, head trauma, central nervous system infections, headaches, and seizures [4]. MRI is a well-tolerated diagnostic modality with no side effects, whereas CT scans expose children to unnecessary radiation, potentially increasing the risk of leukemia and brain tumors [5]. Furthermore, the use of unnecessary imaging tests can have far-reaching consequences, including increased costs and ethical dilemmas arising from incidental findings [6,7]. Despite ongoing research, the prevalence of detecting organic pathology by head imaging in pediatric FEP remains unclear primarily due to limited studies. This study aims to explore current practice patterns related to head imaging in a pediatric FEP population.

## Materials and methods

This study was performed at a community-based health system in the Southeastern United States, which is an 80-bed inpatient behavioral health hospital serving pediatric and adult patients. A retrospective medical record review was performed of all pediatric patients with an encounter of FEP documented between 2013 and 2023, yielding a clustered sample. Inclusion criteria were age less than or equal to 18 years and encounter with the initial application of one of the following principal diagnosis codes: International Classification of Diseases, 10th revision (ICD-10) code F22 (delusional disorders), F23 (brief psychotic disorders), F28 (other nonorganic psychotic disorders), or F29 (unspecified psychosis). Participants from multiple emergency departments (ED) and inpatient psychiatry units were included if they underwent head imaging during the same encounter. Participants were excluded if any of the following data points were missing or incomplete: date of birth, gender, race, ethnicity, health insurance type, admission and discharge dates for the index encounter, hospital location, medications, and CT head or MRI brain imaging date and finding (if performed during the index admission or within one month after discharge).

A manual review of medical records was performed for patients who received a CT head or MRI brain to confirm that the test was performed and determine the indication and interpretation of the test. Descriptive statistics were used to report the study population demographics. An independent t-test (equal variances not assumed) was used for subgroup analysis to compare two independent groups: patients who received head imaging and patients who did not receive head imaging. In this study, patient characteristics (gender, race, ethnicity, health insurance coverage, and prescription medication) were analyzed as categorical variables and compared between the groups. A two-sided p-value of <0.05 was considered statistically significant. Analysis was performed using IBM® SPSS version 29. This study was ruled exempt (category #4) by the health system Institutional Review Board on 7/13/23.

## Results

A total of 113 unique patients met the inclusion criteria for the study. The average age of these patients at the time of the index encounter was 15 and 72/113 (63.7%) were male. Of those reporting race and ethnicity, 63/113 (55.8%) were Black or African American, 39/113 (34.5%) were White or Caucasian, and 7/113 (6.2%) were Hispanic or Latino. The majority (75/113 (66.4%)) were covered under a state-sponsored health insurance plan, 28/113 (24.8%) carried private insurance, 6/113 (5.3%) were uninsured, and 4/113 (3.5%) carried military insurance. See Table 1 for complete demographic information of the study population.

**Table 1:** Study population demographics SD, standard deviation; F22, delusional disorders; F23, brief psychotic disorders; F29, unspecified psychosis; CT, computed tomography

Population	Mean age (years+/-SD)	Gender (n, %)		Race (n, %)		Ethnicity (n, %)		Principal diagnosis code (n, %)		Primary health insurance (n, %)		Psychiatric medications (n, %)	
Total (n=113)	15+/-2.9	Male	72 (63.7%)	African American or Black	63 (55.8%)	Hispanic or Latino	7 (6.2%)	F22	16 (14.2%)	Medicaid	75 (66.4%)	Antidepressants	27 (23.9%)
								F23	27 (23.9%)	Private	28 (24.8%)	Antipsychotics	85 (75.2%)
		Female	40 (35.4%)	White or Caucasian	39 (34.5%)	Not Hispanic or Latino	104 (92%)	F28	2 (1.8%)	Military	4 (3.5%)	Benzodiazepines	5 (4.4%)
		Unknown	1 (0.9%)	Other	11 (9.7%)	Other	2 (1.8%)	F29	68 (60.2%)	Uninsured	6 (5.3%)	Mood stabilizers	18 (15.9%)
												Stimulants	14 (12.4%)
CT head ordered (n=12)	16+/-2.4	Male	8 (66.7%)	African American or Black	10 (83.3%)	Hispanic or Latino	0 (0%)	F22	3 (25%)	Medicaid	9 (75%)	Antidepressants	0 (0%)
		Female	4 (33.3%)	White or Caucasian	2 (16.7%)	Not Hispanic or Latino	12 (100%)	F23	0 (0%)	Private	2 (16.7%)	Antipsychotics	5 (41.7%)
		Unknown	0 (0%)	Other	0 (0%)	Other	0 (0%)	F28	0 (0%)	Military	1 (8.3%)	Benzodiazepines	1 (8.3%)
												Mood stabilizers	0 (0%)
								F29	9 (75%)	Uninsured	0 (0%)	Stimulants	1 (8.3%)

Out of the total study population, 12/113 (10.6%) were confirmed to have received head imaging during or within one month following the index encounter. All patients received CT head and there were no brain MRIs ordered. This subpopulation was composed of a higher percentage of African American or Black patients and none carried the ICD-10 code F23 (acute and transient psychotic disorder), which distinguished these patients from the main study population in which nearly a quarter had this principal diagnosis. They also differed in that a lower percentage were prescribed antipsychotic medications. None of the CT head interpretations revealed significant neurological findings that would suggest a pathophysiological process for psychosis. See Table 2 for detailed information about the patients with a confirmed head imaging study.

**Table 2:** Characteristics of patients with confirmed head imaging study CT, computed tomography; ATV, all-terrain vehicle

Index date	Age (years)	Gender	Reason(s) for CT head order	CT head interpretation	Psychotic symptoms
10/11/13	15	Male	Altered mental status	Normal head CT	Delusions, grandiosity, and disorganized thinking
5/1/14	10	Male	Visual hallucinations	Normal head CT	Visual hallucinations and agitation
9/30/14	17	Male	Mental status changes; hallucinations	Normal head CT	Destroying property, delusions, and visual hallucinations
3/25/15	17	Female	Headaches for 2 weeks	Normal head CT	Paranoia and disorganized thinking
3/17/17	16	Female	Paranoia, delusions, and manic behavior	Normal head CT	Paranoia, delusions, and disorganized behavior
10/10/17	17	Male	Headache; combative	No acute intracranial abnormality; mucosal thickening of right ethmoid air cells and frontal sinus (increased from prior exam)	Agitation, disorganized behavior
10/20/17	17	Male	Altered mental status	Normal head CT	Disorganized behavior and “almost catatonic” behavior
8/16/21	18	Male	New-onset psychosis	No acute intracranial pathology	Acute agitation, disorganized behavior, and delusions
12/8/21	18	Male	ATV accident last week with moderate to severe injury; increasing aggressiveness and paranoid behavior; involuntary commitment	Negative head CT	Aggression, paranoid behaviors, delusions, auditory and visual hallucinations
1/2/22	18	Male	Psychosis	No evidence of acute intracranial abnormality; nonspecific opacification of scattered ethmoid air cells with left frontal sinus air-fluid level (correlate with signs/symptoms of sinusitis)	Disorganized behavior and thoughts, paranoia, delusions, and auditory hallucinations
7/23/22	13	Female	Psychosis	No acute intracranial abnormality seen	Auditory and visual hallucinations, disorganized, bizarre, and manic behavior
1/10/23	15	Female	Altered mental status; manic behavior	No acute intracranial process	Delusions, auditory and visual hallucinations, and manic behavior

When comparing those patients who received head imaging (n=12) to those who did not (n=101), there was no statistically significant difference in the mean number of patients who were male gender (8/12 (66.7%) vs. 64/101 (63.4%) p=0.829), carried Medicaid health insurance coverage (9/12 (75%) vs. 66 (65.4%) p=0.499), or were prescribed an antipsychotic medication (7/12 (58.3%) vs. 78/101 (77.2%), p=0.243). There was a significantly greater percentage of patients who identified as African American or Black (10/12 (83.3%) vs. 53/101 (52.5%) p=0.023) in the head imaging group and none identified as Hispanic or Latino.

## Discussion

This study examined 113 child and adolescent patients meeting the criteria for FEP as characterized by symptoms such as delusions, hallucinations, paranoia, and disorganized thinking or behavior. Only 12 patients underwent a CT head scan and none received an MRI, indicating a notably low utilization of MRI. Among the subset receiving CT head, in addition to psychotic symptoms, four presented with altered mental status, two reported headaches, one had a history of head trauma one week prior to the onset of psychotic symptoms, and one had a comorbid diagnosis of substance use disorder. None exhibited focal neurological deficits during the examination. All patients’ CT scans revealed normal findings, with no acute abnormalities contributing to psychosis. Incidental findings, including left frontal sinus air-fluid levels and mucosal thickening of the ethmoidal and frontal sinus, were observed in two patients, unrelated to psychotic symptoms and requiring no further follow-up. Notably, a higher proportion of Black or African American patients received a CT head scan compared to the overall study population.

When pediatric patients present to the ED or are admitted to an inpatient psychiatric unit with psychotic symptoms, healthcare providers may order routine CT head or MRI scans to rule out any organic pathology or tumors as potential causes. However, the utility of routine head imaging, whether by CT scan or MRI, in cases of FEP remains a topic of debate. Current recommendations for routine head imaging vary among countries, with no consensus on its use in child and adolescent or adult patient populations presenting with psychosis. Some researchers recommend obtaining an MRI in the presence of neurological abnormalities or a history of head injury [8]. The American Academy of Child and Adolescent Psychiatry (AACAP) suggests neuroimaging only when neurological symptoms are evident [9]. Australian and New Zealand clinical practice guidelines recommend a baseline MRI, whereas the British National Institute for Health and Care Excellence (NICE) does not recommend routine CT or MRI scans, advocating for further evidence collection [10,11]. Canadian guidelines suggest ordering head imaging based on history, neurological, and neuropsychological examinations rather than routine use in all patients [12].

Existing data regarding the utility of routine neuroimaging in pediatric patients presenting with FEP is limited but generally aligns with the findings of this study. Cunqueiro et al. (2018) analyzed CT head scans of 362 patients aged five to 20 years and found no actionable acute findings leading them to conclude that routine head CT scans are not indicated in pediatric patients presenting with psychosis in the absence of neurological findings [4]. Similarly, Kular et al. (2021) found no organic causes of psychosis on MRI or CT scans in a study of 34 pediatric patients aged three to 17 years, with four incidental findings on MRI scans [13]. Andrea et al. (2019) also found incidental findings unrelated to psychotic symptoms in a retrospective study of 443 subjects aged 15 to 24 with FEP, suggesting no diagnostic utility of neuroimaging [14]. Another group found a higher prevalence of incidental findings in healthy adults (4.2%) compared to FEP (2.7%), indicating no increased risk of finding significant lesions with routine head imaging in FEP compared to healthy individuals [15].

Studies have consistently shown that head imaging has limited diagnostic utility in patients with FEP, particularly younger patients. Williams et al. (2014) found minimal diagnostic utility of head imaging in younger patients (12-30 years) with FEP, while studies by Bain (1998), Goulet et al. (2009), and LeBaron et al. (2019) spanning decades of practice report limited clinical usefulness of CT or MRI scans among adults with FEP [16-19]. CT scans are faster and less expensive than MRI, but carry the disadvantage of radiation exposure, which can lead to an increased risk of leukemia and brain tumors over time [5]. However, CT head scans may be a better option for individuals with claustrophobia, anxiety, or agitation due to their more open structure. MRI may not be as readily available at every institution, and when they are may require sedation in pediatric patients, potentially increasing the risk of complications and lengthening hospital stays, which further increases economic burdens. Albon et al. (2008) conducted a comprehensive review and economic analysis of neuroimaging in FEP covering both adults and children. Their findings indicated that head imaging, particularly MRI, is not cost-effective and has limited clinical utility in FEP [20].

When comparing the subpopulation of patients who received head imaging compared to the total study population, they tended to be slightly older and were more likely to be Black or African American and carry Medicaid as their primary health insurance. Previous studies have demonstrated that Black or African American patients are more likely to receive a diagnosis of a psychotic disorder than White or Caucasian patients and less likely to get behavioral health care prior to FEP [21,22]. In this study, patients receiving head imaging were less likely to have a principal diagnosis code of F23 (brief psychotic disorders) and instead more likely to have F22 (delusional disorders) or F29 (unspecified psychosis), suggesting that providers may use the latter diagnoses in more atypical or unclear presentations of FEP. The head imaging subpopulation was also less likely to be prescribed any of the psychiatric medications assessed (antidepressants, antipsychotics, mood stabilizers, and stimulants) except for benzodiazepines, which may be a marker of shorter or less complicated psychiatric history prior to FEP. In these cases, providers may consider head imaging in an attempt to rule out another non-psychiatric cause for the patient’s presentation.

Strengths of this study include its multi-location setting, encompassing both EDs and inpatient psychiatric units, and the inclusion of patients over a 10-year period. Limitations include a relatively small sample size and retrospective design. Also, patients with a comorbid diagnosis of substance use disorder were not excluded. Clinicians may be less likely to order head imaging if a patient tests positive for illicit substances, assuming it is the cause of acute psychotic symptoms. Furthermore, patients were not followed to determine if a definitive diagnosis was made after the initial encounter. Despite these limitations, the findings of the present study are consistent with numerous others, which did not identify significant organic findings with substantial effects on clinical management. The findings have implications for reducing unnecessary imaging, improving resource allocation, and enhancing patient safety.

## Conclusions

Routine head imaging may not be warranted in the absence of focal neurological changes, head trauma, or seizures in pediatric patients with FEP. The findings of this study indicate that head imaging typically does not reveal significant pathology that would alter clinical management in this population. Furthermore, this study highlights the preference for CT as the imaging modality, likely due to its expediency. However, it is worth noting that while CT and MRI are comparable in detecting pathology, MRI should be favored whenever indicated and possible, particularly in this patient demographic, to mitigate the risks associated with radiation exposure. Future studies should prioritize prospective designs with larger sample sizes and comparable control groups to better understand the utility of neuroimaging in pediatric FEP.
